# Diversity and Persistence of *Salmonella enterica* Strains in Rural Landscapes in the Southeastern United States

**DOI:** 10.1371/journal.pone.0128937

**Published:** 2015-07-01

**Authors:** John J. Maurer, Gordon Martin, Sonia Hernandez, Ying Cheng, Peter Gerner-Smidt, Kelley B. Hise, Melissa Tobin D’Angelo, Dana Cole, Susan Sanchez, Marguerite Madden, Steven Valeika, Andrea Presotto, Erin K. Lipp

**Affiliations:** 1 Department of Population Health, University of Georgia, Athens, Georgia, United States of America; 2 Department of Environmental Health Science, University of Georgia, Athens, Georgia, United States of America; 3 Warnell School of Forestry and Natural Resources, University of Georgia, Athens, Georgia, United States of America; 4 Centers for Disease and Control and Prevention, Atlanta, Georgia, United States of America; 5 Georgia Department of Public Health, Atlanta, Georgia, United States of America; 6 Department of Infectious Diseases, University of Georgia, Athens, Georgia, United States of America; 7 Department of Geography, University of Georgia, Athens, Georgia, United States of America; 8 Department of Epidemiology and Biostatistics, University of Georgia, Athens, Georgia, United States of America; ContraFect Corporation, UNITED STATES

## Abstract

Salmonellosis cases in the in the United States show distinct geographical trends, with the southeast reporting among the highest rates of illness. In the state of Georgia, USA, non-outbreak associated salmonellosis is especially high in the southern low-lying coastal plain. Here we examined the distribution of *Salmonella enterica* in environmental waters and associated wildlife in two distinct watersheds, one in the Atlantic Coastal Plain (a high case rate rural area) physiographic province and one in the Piedmont (a lower case rate rural area). *Salmonella* were isolated from the two regions and compared for serovar and strain diversity, as well as distribution, between the two study areas, using both a retrospective and prospective design. Thirty-seven unique serovars and 204 unique strain types were identified by pulsed-field gel electrophoresis (PFGE). *Salmonella* serovars Braenderup, Give, Hartford, and Muenchen were dominant in both watersheds. Two serovars, specifically *S*. Muenchen and *S*. Rubislaw, were consistently isolated from both systems, including water and small mammals. Conversely, 24 serovars tended to be site-specific (64.8%, n = 37). Compared to the other *Salmonella* serovars isolated from these sites, *S*. Muenchen and *S*. Rubislaw exhibited significant genetic diversity. Among a subset of PFGE patterns, approximately half of the environmental strain types matched entries in the USA PulseNet database of human cases. Ninety percent of *S*. Muenchen strains from the Little River basin (the high case rate area) matched PFGE entries in PulseNet compared to 33.33% of *S*. Muenchen strains from the North Oconee River region (the lower case rate area). Underlying the diversity and turnover of *Salmonella* strains observed for these two watersheds is the persistence of specific *Salmonella* serovars and strain types that may be adapted to these watersheds and landscapes.

## Introduction

*Salmonella enterica* are zoonotic bacteria associated with a wide range of animals, including humans, where they are a significant cause of enteric disease and often attributed to foodborne transmission [1]. The incidence of salmonellosis has decreased only slightly in the past 26 years (19 cases per 100,000 in 1987 *versus* 15.2 cases per 100,000 in 2013) [2, 3]. While the implementation of HACCP (Hazard Analysis and Critical Control Points) for the food industry in the U.S. has reduced contamination of meats, milk, and eggs with foodborne pathogens [4], there has been increased recognition of *Salmonella* outbreaks associated with fresh produce [5–15]. The cultivation and processing of fruits and vegetables is intimately linked to the environment, where ample opportunities exist to introduce pathogens, through direct contamination from animals in crops and fields and the use of irrigation water that may be contaminated [5, 6, 8, 9, 16]. Although not widely linked to outbreaks, water itself is an important vehicle, especially in sporadic (non-outbreak associated) cases of gastrointestinal illnesses, including salmonellosis [17–19]. Sporadic cases are increasingly associated with a high burden of illnesses and are often not attributable to a particular source, such as food [20]. As a zoonotic agent, animals—livestock as well as wildlife—can be important contributors to the abundance and distribution of *Salmonella* in the environment and possible transmission to humans [21–26]. *Salmonella enterica* is intimately associated with the landscape, and its components (living and non-living). Efforts to examine the environmental ecology of this agent are needed to better understand how it may be controlled, especially in regards to non-foodborne and non-outbreak cases.

The notion that environmental parameters affect both the incidence and distribution of salmonellosis cases is illustrated in part by large regional differences in the rates of reported human cases in the U.S. Variations among states in reported cases are not always explained by differences in surveillance, demographics, patterns in food preparation, or food-distribution networks, suggesting that environmental and ecological factors could affect its relative distribution (e.g., biogeographical patterns [27]). Georgia remains among the states (all in the southeast) with the highest annual prevalence, at 24 cases per 100,000 [28], compared to the national average of roughly 15 cases per 100,000 [2] in the Foodborne Diseases Active Surveillance Network (FoodNet). The prevalence in the southern portion of the state, primarily in the Coastal Plain physiographic province, is markedly higher than that in the northern part of the state’s Piedmont province. In 2011, there were 70.1 cases per 100,000 people in Georgia Public Health District 8–1 and 28.8 cases per 100,000 in Public Health District 10 in 2011, representing the south (Coastal Plain) and north (Piedmont), respectively (GA Dept. of Public Health; dph.georgia.gov). Regional patterns in the epidemiology of salmonellosis are reflected not only by significant differences in reported infection rates, but also in the distribution of specific *Salmonella* serovars between physiographic provinces and individual counties [28].

*Salmonella* is ubiquitous in fresh and marine environmental surface waters [2, 29, 30], but contamination may come from many different routes such as effluent from wastewater treatment plants, contaminated runoff from urban or agricultural areas, overburdened septic systems, or local and migratory fauna [30–34]. Contamination of environmental waters with *Salmonella* may be of a greater public health concern than previously thought due to the ability of it to persist and, in some cases, grow outside of a host organism [29]. This characteristic increases the probability of survival between hosts [35]. The environment, including surface waters, can be considered as a part of the lifecycle of *Salmonella*, and therefore influences the biogeographical patterns of these pathogens.

Previous studies in the Atlantic Coastal Plain (south Georgia and north Florida) indicate that *Salmonella* are commonly detected in the environment, i.e., streams and ponds [36–39]. Frequency of detection ranges from 29% to 96% of samples among these studies, with concentrations reaching 5,400 MPN L^-1^ in the southern reaches of the Upper Suwanee Watershed, which spans southern Georgia and north Florida [36, 39]. At the uppermost reaches of the Upper Suwanee Watershed in south Georgia, the Little River is typical of the heavily vegetated, slow-moving stream systems in this region. We have previously shown that 79% of sites along the Little River were positive for *Salmonella* [37] with levels ranging from 2.5 MPN L^-1^ to 36.3 MPN L^-1^. *Salmonella* densities are positively impacted by precipitation and temperature (e.g., summer season) when serovars associated with human cases are also more likely to be detected [38]. Here we expanded on this work and examined the distribution and diversity of *Salmonella* serovars and strains across geographic space, time, and between water and wildlife reservoirs, which may affect exposure routes and transmission to humans.

## Material and Methods

### Description of study sites

**Fig 1:**
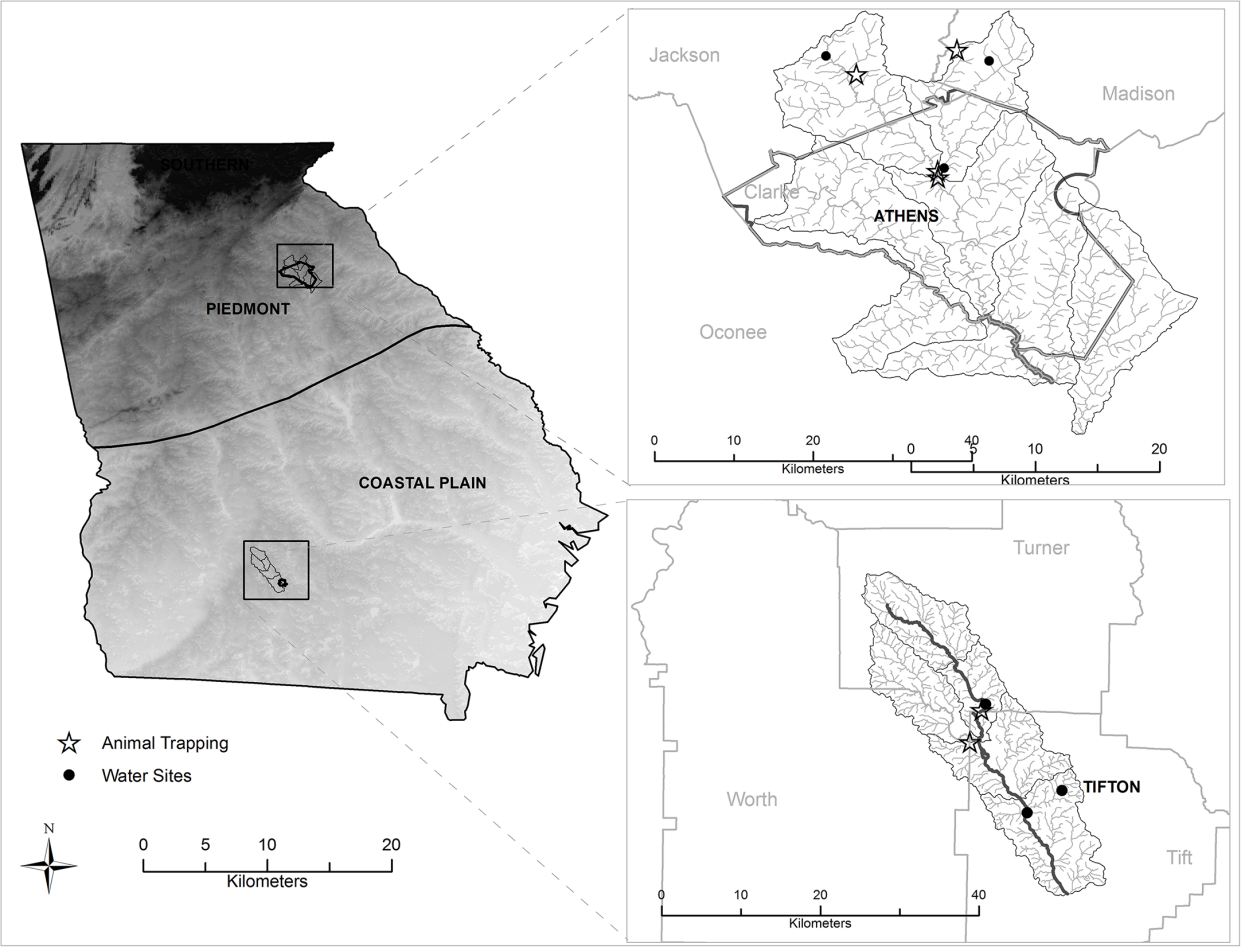
Map of sampling areas in the Oconee River watershed (near Athens in the Piedmont physiographic province) and Little River watershed (near Tifton in the Coastal Plain physiographic province). Base map source: U.S. Geological Survey, Department of the Interior. (http://water.usgs.gov/lookup/getspatial?physio). Background: watershed produced using ESRI-ArcGIS (LM_LICENSE_FILE: 1700@wrrs.gly.uga.edu) based on U.S Geological Survey, National Elevation Dataset (NED), 2012. Site location: Department of Environmental Health Science-UGA. (Produced by Presotto A, 2015).

The sample areas were located in two distinct physiographic regions of Georgia, USA—the low lying Coastal Plain and the higher elevation Piedmont physiographic province (Fig 1). The regions were also distinguished by prevalence of salmonellosis. Otherwise the selected areas were similar in watershed size, land use, population, and median incomes.

The 334 km^2^ Little River watershed is near Tifton, Georgia in the South Atlantic Coastal Plain, and forms the headwaters for the larger Upper Suwannee Watershed. The Little River watershed is typified by broad floodplains with very poorly defined stream channels and gently sloping uplands. Approximately 45% of the watershed is woodland, 37% crops, 4% pastures, 7% idle, and 7% roads, urban, and water (as described in [37]). Swamp hardwoods occur along the stream edges and are often accompanied by thick undergrowth forming the riparian vegetation boundary along stream networks. Three sampling stations were selected representing first to fourth stream orders with varying levels of flow throughout the year. First order streams are headwaters and are small and narrow whereas fourth order streams are fed by multiple tributaries and are larger and broader. Stations were located in Tift County (upstream of the City of Tifton) in GA Public Health District 8–1. Tift County is rural but is considered to be urbanizing. The 2010 population was 40,118 (59.8 people km^-2^) with 6.9% of the population under the age of 5 [40]. The per capita income was $18,928 [40]. This district has the highest case rates for salmonellosis in the state (70.1 cases per 100,000 in 2011).

The Oconee River Basin consists of two headwater tributaries, the North Oconee River and the Middle Oconee River, which originate at the northern end of the basin in the Piedmont Upland physiographic province, at an elevation of about 305 m above mean sea level. These headwater streams are generally well entrenched, flow through narrow floodplains, and have steep gradients ranging from 0.15 to 1.4 m km^-1^. These rivers flow for approximately 100 km to a point just south of Athens, Georgia, where they join to form the Oconee River. Land use in the upper portion of the basin is primarily rural, with poultry farming, dairy farms and grazing for beef production as primary uses. Three sampling stations were selected along the North Oconee River and its tributaries in Jackson County, upstream of the city of Athens, and included first to fourth ordered streams. Jackson County recorded a population of 60,485 in 2010 (68.8 people km^-2^) with 6.8% of the population under the age of 5 [40]. The per capita income was $22,830 [40]. Jackson County is located in GA Public Health District 10, which is a lower case rate area for salmonellosis in Georgia (28.9 cases per 100,000 in 2011).

### Sample collection

Water samples were collected monthly from December 2010 to November 2011 at each of the six stations (Fig 1), which were accessible on foot with public access. Sterile 1-L polypropylene bottles were filled by hand at the deepest part of the stream. For a limited number of collection events (n = 3 in each watershed), samples were obtained from well water, after flushing the spigot for 5 min, at farms near the sampling sites (in coordination with owners). All samples were maintained on ice, transported to the laboratory, and processed within 6 h of collection. Wildlife near each of the six sites was also sampled. Songbirds, raccoons, and opossums were the focus of the active surveillance. Mist nets were used as previously described [41] to capture birds. Birds were held in individual disposable paper bags until they defecated. After 1 h, even if the bird had not defecated, it was released to avoid capture-related mortality. Opossums and raccoons were captured with baited live box traps (Havahart, Woodstream Corp, Lititz, PA). Briefly, traps baited with sardines were deployed at sunset and checked at dawn. If an animal was captured, the trap was turned so that it would stand vertically and the animal was gently forced to the bottom and hand injected with an anesthetic. Once the animal had reached a light plane of anesthesia, it was removed from the trap, and approximately 1 g of feces was removed directly from the rectum by digital extraction. Fresh fecal material was immediately immersed in 10 ml of dulcitol selenite broth and maintained at room temperature until submitted to the Athens Diagnostic Laboratory for isolation. Samples were submitted to the laboratory within 48 h. The University of Georgia Animal Care and Use and Procedures Committee approved protocols involving capture and handling of animals associated with this project (AUP # A2010 08-159-Y3-A0).

### *Salmonella* isolation

Each water sample (≤100 ml) was filtered in duplicate onto 0.45 μm 47-mm mixed cellulose ester membranes, inserted into a sterile 50-ml centrifuge tube containing 20 ml of 1% sterile peptone broth and incubated overnight at 37°C. A 100-μl aliquot of turbid broth culture was used to inoculate a15-ml tube 10 ml Rappaport-Vassiliadis (RV) broth, which was then incubated at 37°C for ~24 h. One loopful of overnight growth from RV broth was spread onto XLD agar plates and incubated for 24 h. Presumptive *Salmonella* colonies (H_2_S positive) were picked and transferred to LB agar stabs. Cultures were streaked for isolation three times before final *Salmonella* confirmation and serovar determination (see below). Each sample was scored as positive or negative for *Salmonella* presence following confirmation steps.

For animal feces, Dulcitol Selenite (Difco; Detroit, MI) was inoculated with fecal samples and incubated at 42 ± 0.5°C. A 10 μl loopful of overnight growth from enrichment broth was streaked onto a XLT4/BGN bi-plate (Remel Inc.; Lenexa, KS) followed by 37°C overnight incubation [42, 43].

All H_2_S positive colonies were further characterized biochemically to identify *Salmonella*. Real time PCR was used to screen Selenite broths that were culture negative for *Salmonella* [44]. PCR template was prepared from a pool of three Selenite broth cultures. DNA was isolated from 1 ml of the pooled enrichment using Ultra Clean Fecal DNA Kit (MO Bio Inc., Carlsbad, CA). Positive pools were then tested individually by PCR and subcultured simultaneously onto *Salmonella* selective media. A delayed secondary enrichment was done for culture negative, PCR positive Selenite enrichment broths [43]. Any suspect, biochemically atypical *Salmonella* were confirmed by PCR [44]. *Salmonella* isolates were forwarded to the National Veterinary Service Laboratory (Ames, IA) for serotyping.

### Molecular typing of *Salmonella* isolates by pulsed-field gel electrophoresis

Pulsed-field gel electrophoresis (PFGE) was used to determine the genetic relatedness [45–47] among *Salmonella* isolates obtained during this study, archived isolates from environmental and animal sources, and human isolates represented in the PulseNet USA national database, which included reports from states in the southeast US (GA, FL, SC, and AL). A master database of *Salmonella* PFGE patterns was generated in BioNumerics (Applied Maths; Austin, TX). Comparisons were made between PFGE patterns using Dice coefficient [48] and unweighted pair group method of arithmetic averages (UPGMA) clustering. Clusters were identified based on a 75% similarity cut-off.

## Results

### Serovar distribution in *Salmonella* isolated from the Little River and Oconee River Basins

In total, 1,029 isolates from the two watersheds (water and animals) and archived environmental and animal samples were processed for serovar and PFGE pattern. These included 355 isolates from water and animals collected in the Little River (Upper Suwannee) and Oconee River watersheds (2005–2011) and an additional 674 archived isolates from animal sources (various species) obtained from the *Salmonella* Reference Collection (SARA 1–72) [49] and past studies [21, 37, 48, 50–53]. Thirty-seven unique serovars were identified from salmonellae isolated from water and animals in the Little River and Oconee River watersheds. These included 15 serovars that were ranked among the top 20 for human cases in the US and in Georgia [31]. Eighteen serovars were recovered only from water (represented by 53 isolates) and included six of the top 20 ranked serovars in human cases (U.S. and Georgia). Only two serovars, Braenderup and Paratyphi B var L (+) tartate +, were found in both watersheds and none of the PFGE types were shared between the two regions (Table 1). Only five serovars were recovered solely from animals (cattle, hogs, opossum, raccoons or song birds) and none included serovars commonly associated with human cases. Two serovars were found in animals from both watersheds (Dublin and Muenster) (Table 1). Most isolates (82%; 291/355) were associated with serovars found in both water and animals, including 4 isolates from shallow wells in the Little River watershed. In all, 14 serovars were recovered from both sources and nine of these were among the top 20 for human cases (US and Georgia) (Table 1). Unlike water-only and animal-only serovars, most of the serovars from both sources were also found in both watersheds (11 were shared).

**Table 1:** *Salmonella* serovars isolated from the Little River and Oconee River watersheds (2005–2011).

Source	Serovar	Little River		Oconee River		Both watersheds
		# of isolates	# PFGE types	# of isolates	# PFGE types	# isolates with shared PFGE type^a^
Water	16:z:10	1	1	0	0	0
Only	30:-:lw	1	1	0	0	0
	47:z4z23:-	3	1	0	0	0
	***Braenderup***	13	3	2	2	0
	***Enteritidis***	2	2	0	0	0
	***Heidelberg***	0	0	1	1	0
	***Infantis***	0	0	1	1	0
	Kentucky	4	0	0	0	0
	Kiambu	0	0	1	1	0
	Liverpool	2	2	0	0	0
	Livingstone	0	0	1	1	0
	Oranienburg	0	0	3	2	0
	Ouakam	0	0	1	1	0
	**Paratyphi B** ^b^	3	1	5	2	0
	Senftenberg	0	0	3	2	0
	Tamberma	1	1	0	0	0
	Thompson	0	0	4	1	0
	***Typhimurium***	0	0	1	1	0
Animal	III 44:z4, z32:-	1	1	0	0	0
Only	O-:lz4, z23:-	1	1	0	0	0
	Arizona	1	1	0	0	0
	Dublin	3	1	1	1	4
	Muenster	3	1	1	1	4
Water +	IV 40: z4: 32	1	1	3	1	0
Animal	***Anatum***	6	4	1	1	0
	***Bareilly***	12	5	7	3	0
	Gaminara	8	7	2	1	0
	Give	27	7	15	13	15
	*Hartford*	16	8	21	2	0
	*Inverness*	2	2	0	0	0
	Mbandaka	9	2	3	2	0
	Meleagridis	15	4	0	0	0
	***Montevideo***	5	3	4	3	0
	***Muenchen***	11	10	36	25	5
	***Newport***	2	1	6	5	0
	*Rubislaw* ^c^	41	36	29	14	4
	***Saintpaul***	9	4	0	0	0

Serovars noted in bold are those ranked among the top 20 in human cases for the US (2009–2011); serovars in italics are those ranked in the top 20 in human cases in Georgia (31).

^a^ Isolates shared one PFGE type (per serovar)

^b^ Paratyphi B var L (+) tartate +

^c^ Included one isolate collected from shallow well water in the Little River watershed

Regardless of source, *Salmonella enterica* Rubislaw, Give, Hartford, Braenderup, and Muenchen were the serovars most frequently isolated from either region, accounting for 53% and 62% of total isolates from the Little River and Oconee River watersheds, respectively. *Salmonella* Muenchen and Rubislaw were the most frequently encountered serovars in both river basins. With the exception of *S*. Braenderup, most other *Salmonella* serovars encountered in both watersheds were transient, being isolated only once for a given year.

### Strain distribution in *Salmonella* isolated from the Little River and Oconee River Basins

**Fig 2:**
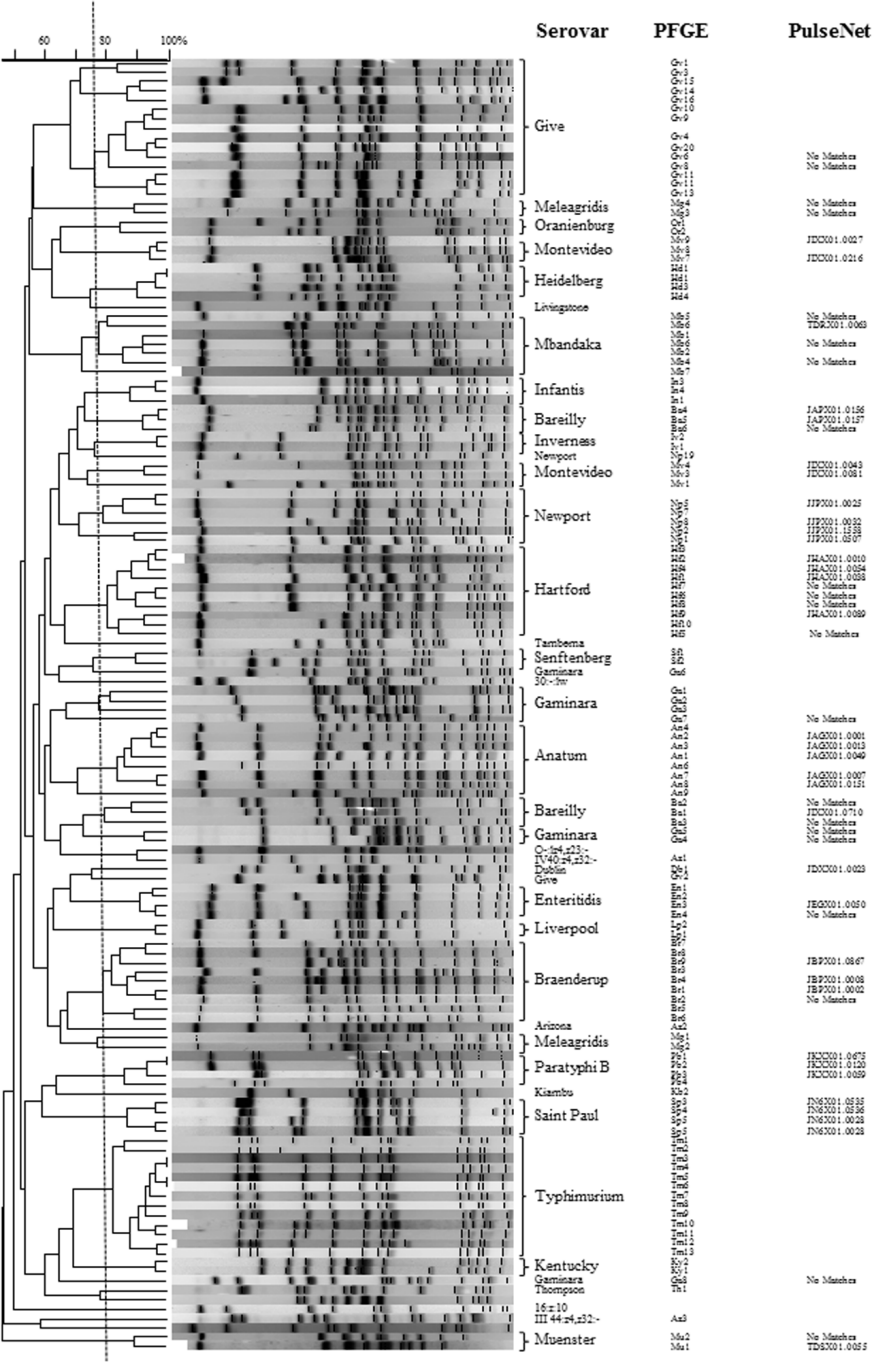
Dendrogram of representative *Salmonella* PFGE patterns for 37 *Salmonella* serovars (excluding *S*. *enterica* serovars Muenchen and Rubislaw) collected from Oconee and Little River watersheds and archived isolates with similar PFGE profiles. *Salmonella* PFGE patterns generated in this study were compared to a BioNumerics database of PFGE entries of *Salmonella* isolates from various animal species and to the CDC PulseNet data base of isolates from human cases. Vertical line indicates 75% similarity.

**Fig 3:**
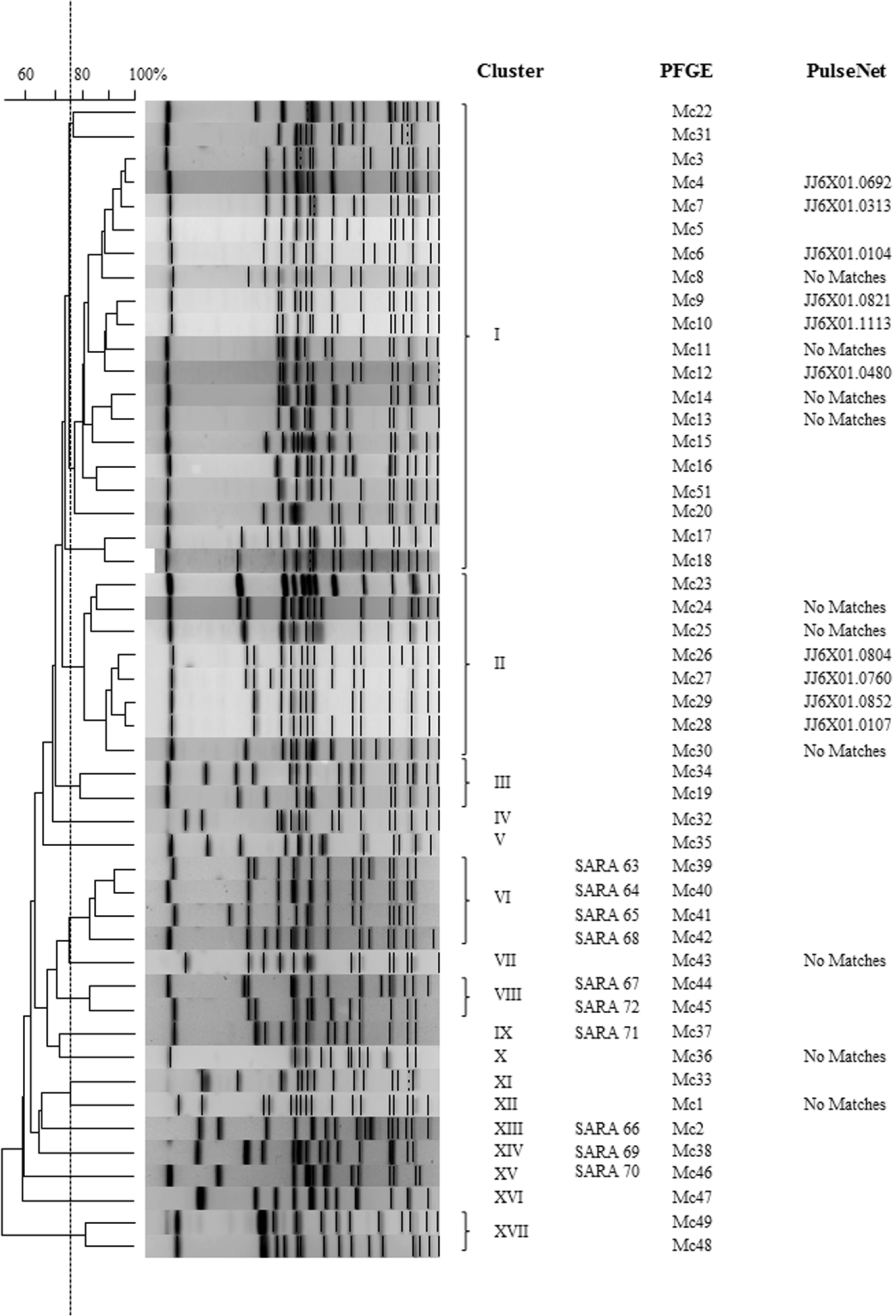
Dendrograms of representative *Salmonella* PFGE patterns for *Salmonella* serovars Muenchen collected from Oconee and Little River watersheds and archived isolates with similar PFGE profiles. Vertical line indicates 75% similarity.

**Fig 4:**
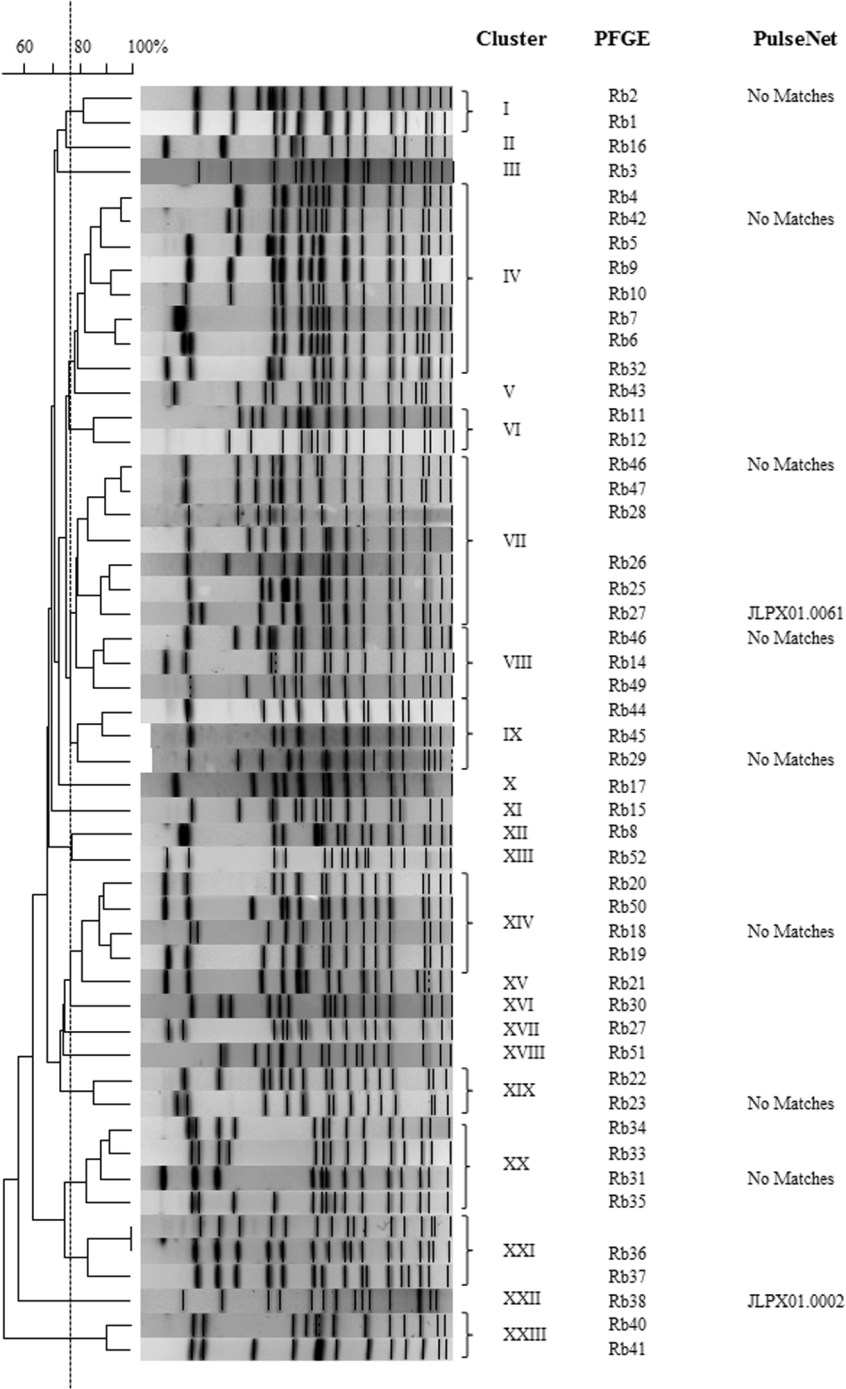
Dendrograms of representative *Salmonella* PFGE patterns for *Salmonella* serovars Rubislaw collected from Oconee and Little River Basins and archived isolates with similar PFGE profiles. Vertical line indicates 75% similarity.

*Salmonella* PFGE patterns for environmental and archived isolates were compared to each other to evaluate trends in strain persistence and relatedness from the two sample sites. In addition to patterns in serovar distribution (Table 1), there was also considerable strain diversity in *Salmonella* isolated from the two study sites. Among 37 *Salmonella* serovars isolated from either region, there were 204 unique PFGE patterns for the water and animal isolates analyzed. Frequently PFGE patterns clustered together by serovar (≥75% similarity) (Fig 2); however, there were several *Salmonella* serovars where PFGE patterns with <75% similarity generated two or more clusters (*S*. Bareilly- 2 clusters; *S*. Gaminara- 6 clusters; *S*. Give- 4 clusters; *S*. Meleagridis- 2 clusters). Two *Salmonella* serovars, *S*. Muenchen and *S*. Rubislaw, exhibited the greatest diversity in PFGE patterns, necessitating separate cluster analyses (Figs 3 and 4). *Salmonella* Muenchen PFGE patterns fell into one of 16 clusters; clusters I & II represented 41% and 16% of PFGE profiles, respectively. *Salmonella* Rubislaw demonstrated even greater diversity in PFGE profiles, with patterns falling into one of twenty-three clusters. No single *S*. Rubislaw PFGE cluster accounted for more than 15% of the total patterns. While there was a high level of diversity in PFGE profiles for both serovars, more patterns matched human clinical cases entered in PulseNet for *S*. Muenchen (44%; n = 25) than for *S*. Rubislaw (18%; n = 22).

While there were several *Salmonella* serovars common to both watersheds, only 5 of 204 PFGE types observed were shared among isolates from the two regions (Table 1). Most *Salmonella* PFGE types (82%) were rare, only appearing once. There were 11 unique PFGE types that were identified in isolates from both water and animals within the same watershed sampling area (Table 2). For eight of these, water and animal isolates were collected within the same season or year and included *Salmonella* serovars *S*. Anatum, *S*. Bareilly, *S*. Give, *S*. Hartford, *S*. Mbandaka, *S*. Montevideo, and *S*. Newport. Most PFGE types were specific to water or to animals.

**Table 2:** *Salmonella* strains (PFGE types) present in both animals and water of the Little River or North Oconee River watersheds (Georgia, USA).

Serovar	Watershed	# of Strains	Sources	Season/Year Collected
Anatum	Little River	1*	Water	Winter 2011
			Opossum	Spring 2011
Barielly	Little River	1*	Water	Spring 2005, Summer 2007, Fall 2011
			Raccoon	Summer 2011
	Oconee River	1	Opossum	Fall 2010, Summer 2011
			Water	Summer 2011
Give	Little River	1	Opossum	Fall 2010, Winter, Spring, Summer, Fall 2011
			Water	Fall 2011
Hartford	Oconee River	1*	Raccoon	Winter and Spring 2011
			Water	Summer and Fall 2011
Mbandaka	Little River	1	Songbird	Spring 2011
			Opossum	Summer 2011
			Water	Fall 2011
Montevideo	Little River	1*	Water	Winter and Fall 2011
			Raccoon	Winter 2011
Muenchen	Oconee River	1*	Water	Spring 2005
			Opossum	Summer 2011
Newport	Little River	1*	Raccoon	Winter 2011
			Water	Summer 2011
Rubislaw	Oconee River	2	Water	Spring 2005
			Opossum	Winter, Summer, Fall 2011

* indicates PFGE pattern matching isolate in CDC PulseNet database. [Winter (Jan, Feb, Mar), Spring (Apr, May, Jun), Summer (Jul, Aug, Sep), and Fall (Oct, Nov, Dec).]

Despite turnover of *Salmonella* strains, there were several *Salmonella* strains isolated at least twice from either watershed, representing 19 serovars and 34 PFGE types. Most of the PFGE types were encountered sporadically, often isolated once in a given year. Just over half (18/34) were encountered multiple years; 14 that were originally isolated in 2005 were still present in 2010 and 2011 (4 PFGE types in the Little River and 10 in the Oconee River) (Table 3). Only two PFGE types were consistently present between 2005 and 2011; *S*. Braenderup type Br1 and *S*. Saintpaul type Sp5 were isolated in the Little River in 4 and 3 separate years, respectively.

**Table 3:** *Salmonella* serovars and PFGE types isolated multiple times in the Little River or Oconee River watersheds (both water and animal sources) between 2005 and 2011.

Serovar	PFGE type	PulseNet ID	Watershed	Source	Date of Collection
47:z4z23:-	Tb1	Not determined	Little River	Water	Feb, Mar, Dec 2007
Anatum	An2	JAGX01.0001	Little River	Water	Mar 2011
				Opossum	Apr 2011
	An7	JAGX01.0007	Little River	Water	Jun, Aug 2007
Bareilly	Ba1	JIXX01.0710	Oconee River	Water	Sep, Oct 2011
	Ba2*	No matches	Oconee River	Water	Aug 2011
				Opossum	Dec 2010, Sep 2011
	Ba4*	JAPX01.0156	Little River	Water	May, Sep 2005, Oct 2011
				Raccoon	Aug 2011
	Ba5	JAPX01.0157	Little River	Water	Feb, Jun 2011
	Ba6	No matches	Little River	Water	Aug, Sep 2005
Braenderup	Br1*	JBPX01.0002	Little River	Water	May, Jul, Aug 2005; Jan 2006; Jun, Jul, Aug, Sep 2007; Feb 2011
Dublin	Db1*	JDXX01.0023	Little River	Songbird	Dec 2010, 2011
Gaminara	Ga4*	No matches	Little River	Water	Aug 2005, Feb 2007
Give	Gv4*	No matches	Little River	Opossum	Dec 2010; Feb, May Aug, Oct, Nov 2011
	Gv11*	No matches	Oconee River	Water	Apr 2005, Feb 2011
Hartford	Hf1*	JHAX01.0038	Oconee River	Water	Apr 2005, Feb 2011
	Hf2	JHAX01.0010	Oconee River	Water	Jun, Nov 2011
				Raccoon	Feb, Apr, Jul 2011
	Hf9	JHAX01.0089	Little River	Water	Jun, Aug, Nov 2005
Mbandaka	Mb3	No Matches	Little River	Water	Oct 2011
				Songbird	Apr 2011
				Opossum	Aug 2011
Meleagridis	Mg3	No matches	Little River	Water	Jan, Feb, Mar, Oct 2011
	Mg4	No matches	Little River	Water	Feb, Sep 2007
Muenchen	Mc4*	JJ6X01.0692	Oconee River	Water	Apr 2005
				Opossum	Jul 2011
	Mc24*	No matches	Oconee River	Water	Apr 2005, Jun 2011
	Mc28	JJ6X01.0107	Little River	Water	Aug, Nov 2005
Montevideo	Mv3	JIXX01.0081	Little River	Water	Mar, Oct 2011
				Raccoon	Feb 2011
Newport	Np5*	JJPX01.0025	Oconee River	Water	Apr 2005, Mar 2011
	Np8	JJPX01.0032	Little River	Water	Sep 2011
				Raccoon	Feb 2011
Paratyphi B	Pb3*	JKXX01.0059	Little River	Water	Nov 2005, Jun 2007
			Oconee River	Water	Apr 2005, Jan 2011
Rubislaw	Rb20*	JLPX01.0108	Little River	Water	Jul, Dec 2005; Jan 2011
	Rb27*	JLPX01.0061	Oconee River	Water	Apr 2005, May 2011
	Rb34*	No matches	Oconee River	Water	Apr 2005
				Opossum	Feb, Aug, Nov 2011
	Rb36*	No matches	Oconee River	Water	Apr 2005
				Opossum	Feb, Mar 2011
Saintpaul	Sp5*	JN6X01.0028	Little River	Water	Jul 2005; Sep, Nov 2007; Feb 2011
Senftenberg	Sf2*	Not determined	Oconee River	Water	Dec 2010, Oct 2011
Thompson	Th1	Not determined	Oconee River	Water	Apr 2005; Sep, Oct 2011

*isolated in multiple years

### Distribution of *Salmonella* strains associated with human illness in the Little River and Oconee River watersheds

The incidence of salmonellosis in Georgia is skewed within the state, with the highest incidence occurring in the southern region. To address whether the distribution of specific serovars or strains in the environment might be associated with these trends in human cases, select *Salmonella* PFGE types identified between Little River and Oconee River isolates were compared to CDC PulseNet database for matching PFGE patterns among human isolates. Due to the high number of PFGE patterns identified, only a subset were compared, representing 1) the most common *Salmonella* serovars in human cases, 2) a *Salmonella* strain that was persistent in either watershed, or 3) present in both water and wildlife from that locale (n = 113). Approximately, half of the *Salmonella* isolated from water and wildlife sources had matching PFGE patterns with PulseNet database of human isolates (Table 4). Human cases from the state of Georgia were reported for 7 and 4 of the *Salmonella* PFGE types from Little River and Oconee River watersheds, respectively (represented by serovars: *S*. Anatum, *S*. Braenderup, *S*. Hartford, *S*. Montevideo, *S*. Muenchen, *S*. Newport, *S*. Paratyphi B, and *S*. SaintPaul). There was no significant difference in preponderance of *Salmonella* strains that had matching PFGE patterns with human isolates in PulseNet between the two river basins (Table 4); however, there was a difference in the distribution of *S*. Muenchen strains associated with human cases. Ninety percent of *S*. Muenchen strains (n = 10) from the Little River had matching PFGE profiles with PulseNet entries, while only one third of *S*. Muenchen PFGE patterns for Oconee River isolates (n = 24) had matches with PulseNet database.

**Table 4:** *Salmonella* PFGE types isolated from the Little River and Oconee River watersheds associated with human illnesses.

	Little River	Oconee River
Total PFGE types submitted to CDC PulseNet	75	40
PFGE types with matches to patterns in PulseNet	33	19
#Total isolates with PFGE pattern matching PulseNet database (all serovars)	74 (46%^b^; n = 161)	49 (50%^b^; n = 98)
• Muenchen isolates	9 (90%^c^; n = 10)	8 (33%^c^; n = 24)
• Rubislaw isolates	5 (21%^c^; n = 24)	2 (7%^c^; n = 29)
PFGE types associated with illnesses in Georgia ^a^	7	4
#Cases in Georgia associated with matching PFGE types ^a^	28	32

^a^ Of the PulseNet matches, the search of database was restricted to year of isolation for environmental strain

^b^ Of PFGE patterns submitted to PulseNet, proportion of isolates with PulseNet matches

^c^ For *S*. Muenchen or *S*. Rubislaw PFGE patterns submitted to PulseNet, the proportion of isolates with PulseNet matches

## Discussion

The overall *Salmonella* serovar composition noted in this study and in prior work was similar between these two rural watersheds [36, 37, 53]. *Salmonella* serovars generally associated with food animals were rare in both watersheds (e.g., *S*. Newport, *S*. Enteritidis, *S*. Typimurium) [54–56], whereas elsewhere in the United States and Canada, such *Salmonella* serovars have been frequently isolated from watersheds [57, 58]. The serovar composition found in the present study also differed from the findings in agricultural ponds within the coastal plain of Georgia, where serovars associated with food production were common and displayed a relatively low diversity of serovar type [39]. In the natural and flowing river systems that were the focus of this study, serovar diversity was high and only two serovars common in food were observed, and were found only rarely (*S*. Newport and *S*. Saintpaul) [59, 60]. Here, *S*. Muenchen and *S*. Rubislaw were the most commonly detected isolates in this study, with 47 and 70 isolates, respectively. These serovars have also been found in other natural waters [53, 61], including other areas of the Atlantic Coastal Plain [36, 53, 61]. These serovars are also often associated with human cases in southern Georgia, where they ranked 4^th^ and 11^th^ in reported cases between 2000 and 2006 (Georgia Dept. of Health; District 8–1). Interestingly, common poultry *Salmonella* serovars such as *S*. Enteritidis and *S*. Heidelberg [62] were very rarely isolated even though north Georgia has the greatest concentration of poultry production in the state [63] and application of poultry manures to fertilize pasture land is a common practice in many areas of the state [38, 64].

Fourteen of the 37 serovars identified in this study, representing 82% of the isolates, were recovered both from surface waters and wildlife captured nearby (one serovar, Rubislaw, was also identified from well water). Eleven PFGE types were identical between water and animals. In contrast, only five serovars were recovered from animals alone. While 18 serovars were found solely in water, these represented only 15% of the isolates. *Salmonella* isolation was especially common in opossums and raccoons caught in proximity to the water collection sites in both watersheds. It is unknown how much these animal species and others [65, 66] contribute to *Salmonella* loading in either river basin, but the results indicate that a significant population of *Salmonella* strains may be moving between wildlife hosts and the environment, including water.

Interestingly, many of the *Salmonella* serovars identified in the Little River (southern Georgia) have been associated with outbreaks epidemiologically linked to fresh produce [15]. There is a significant delineation in agricultural output within the state of Georgia, where the southern region is noted more for produce production. As water is especially important in cultivation and production of produce, the watersheds in southern Georgia are potentially important conduits for introducing *Salmonella* contamination to food products. Irrigation ponds in the region have been shown to harbor *Salmonella*, but at a lower diversity than noted in the watershed studies presented here [39]. Differences between a pond environment and a flowing river system may reflect differences in loading and contamination and may also reflect ecological differences in the system.

The overall level of *Salmonella* and their diversity in natural watersheds supports the idea that landscapes may be an important feature in sporadic transmission to humans. Similar disparities in the geographic distribution of human illnesses associated with other zoonotic bacteria such as *Escherichia coli* O157:H7 and *Campylobacter* sp. have been observed elsewhere, where higher illness rates are found in regions with high livestock densities, likely due to increased animal contact and environmental exposure [18, 19, 67, 68]. Evidence from recent work suggests that direct environmental exposures may be important in non-outbreak scenarios. For example, the prevalence of private wells for drinking water and use of septic systems are noted risk factors for non-outbreak associated salmonellosis, especially among children [17, 20]. Similar risk factors were also noted for specific serovars common in the southeast U.S. [20] While both of the rural areas investigated in this study rely on septic systems for waste disposal, there is a higher rate of private (untreated) wells as the source of drinking water in the southern part of the state (28.7% of the total population and 95% of the rural population [69].

*Salmonella enterica* is a commonly detected pathogen in the waters of Georgia, and here showed a very high level of diversity both at the serovar and PFGE-type strain level, especially in the Little River watershed in the Atlantic Coastal Plain. Similar observations have been made regarding the genetic diversity of *Salmonella* isolated from other river systems [58], including the Suwanee watershed of South Georgia/North Florida [36, 58]. This temporal turnover of *Salmonella* strain types in the two river basins may follow point-source contamination and possible impact of land use on prevalence and diversity within these environs. While there was significant genetic diversity in each of these two river basins, we did identify matching PFGE patterns between *Salmonella* recently isolated from the Oconee River and Little River and those from earlier studies of the two watersheds [37, 53]. This suggests certain *Salmonella* strains persist in this environment due to either continued contribution by an animal population or long-term persistence within this aquatic environment, suggesting a specific niche within the river or watershed. For example, evidence indicates that sediment may support long-term survival and persistence for *Escherichia coli* O157:H7 [70], *Salmonella* [70, 71], and *Campylobacter* [72]. Storm events can churn up this sediment and reintroduce these dormant, persistent *Salmonella* strains into the water column. *Salmonella* levels within the water column significantly increase in the river following storm events and there is a positive correlation in *Salmonella* prevalence, concentration, and rainfall [37].

Over all serovars tested, there were no differences between the Oconee River and the Little River basin in the percentage of environmental *Salmonella* strains that matched human clinical strains (~50% each, determined by identical PFGE patterns with human isolates reported to the PulseNet database), despite the fact that prevalence of salmonellosis is higher in the south (including the Little River area). When focusing on specific *Salmonella* serovars, we identified distinct differences between the watersheds in the proportions of strains matching those of human isolates in PulseNet. For example, 90% of the *S*. Muenchen isolates from the Little River watershed matched entries in PulseNet versus only 33% from the Oconee River watershed. The greater preponderance of matches between environmental and human isolates for the Little River and the Oconee River is especially significant due to the high level of genetic diversity inherent in these populations and may suggest differences in climate, landscape, and human activities between the two watersheds. In addition to epidemiological studies that may help to determine where and how humans may be exposed through the environment (including water), in the future whole genome sequencing of these environmental, animal, and human isolates, with matching PFGE patterns, will allow us to discern genetics that underlie the pathogenic potential of environmental *Salmonella* and genetic markers that identify point source for contamination.

## Conclusion

Studies examining risk factors for salmonellosis can no longer focus only on impacts associated with food production. *Salmonella* is a broad zoonotic agent that is likely part of the ecology of the landscape, with high rates of exchange probable between humans, water, and wildlife. There are several inherent differences between North and South Georgia in its geography, geology, land use, and ecology that may be driving the rates of salmonellosis within the state. The recent publication of the *Salmonella* Atlas for 32 major *Salmonella* serovars by the CDC further supports geographical differences in the incidence of disease in the United States [73]. Understanding ecological interactions between pathogens, the environment, and humans is essential for reducing the burden of human illnesses due to *Salmonella*.
